# Treatment of Chronic Kidney Disease: Moving Forward

**DOI:** 10.3390/jcm11236948

**Published:** 2022-11-25

**Authors:** Giacomo Garibotto, Daniela Picciotto, Pasquale Esposito

**Affiliations:** 1 Department of Internal Medicine, University of Genoa, DiMI, 16132 Genoa, Italy; 2 IRCCS Ospedale Policlinico San Martino, Clinica Nefrologica, Dialisi, Trapianto, 16132 Genoa, Italy

Chronic kidney disease (CKD) affects ~10% of the adult population [1]. Data from national and international databases show a continuous growth in the incidence and prevalence of CKD, a finding which underscores the ineffectiveness of current policies and strategies for its prevention and treatment [2]. The new epidemics of obesity and diabetes, through their negative effects on the kidney as well as associated morbidity, are focal points of the global increase in CKD. In turn, CKD drastically increases the risk for cardiovascular morbidity and mortality [1,2,3]. From a theorical standpoint, effectively treating CKD would imply slowing disease progression, preventing the development of end-stage kidney disease (ESKD) and cardiovascular complications, thereby increasing survival.

Preservation of kidney function can be achieved through both non-pharmacological strategies and CKD-targeted pharmacological interventions [4]. For decades, treatment of CKD has included targeting blood pressure and albuminuria, nutritional intervention, avoiding potential nephrotoxins and obesity, drug dosing adjustments, and cardiovascular risk reduction. However, despite these approaches, the number of patients reaching ESKD is still increasing and the death rate is exceedingly high [3,4,5].

Luckily, nephrology is a fast-moving science. The novel obesity and diabetes pandemic has created the conditions for rapid knowledge generation. An example of the new knowledge is offered by the new treatments of diabetic kidney disease (DKD). Until recently, prevention of DKD progression was based around strict blood pressure (BP) control, using renin–angiotensin system blockers that simultaneously reduce BP and proteinuria, adequate glycemic control and control of cardiovascular risk factors. New drugs which modify intrarenal haemodynamics (such as renin–angiotensin (RAS)–aldosterone pathway modulators and sodium–glucose cotransport inhibitors (SGLT2i)) can preserve the kidney from damage by decreasing intraglomerular pressure independently of blood pressure and glucose control, whereas other novel agents (such as mineralocorticoid receptor antagonists—MRA) offer kidney protection in proteinuric patients through their antifibrotic mechanisms. The new guidelines have now included as a first-line therapy the use of SGLT2i in addition to RAS inhibition, and as an additional risk-based therapy, glucagon-like peptide 1 receptor antagonists (GLP1-RA) and non-steroid MRA [4].

In addition, a couple of years ago, the Dapagliflozin and Prevention of Adverse Outcomes in Chronic Kidney Disease (DAPA-CKD) trial [6] demonstrated that the SGLT2i dapagliflozin reduced the progression of CKD with proteinuria in patients with or without diabetes. As the DAPA-CKD trial included many patients with immunoglobulin A nephropathy (IgAN), dual renin–angiotensin/SGLT2 inhibition may become a new standard of treatment for this common disease [7]. In addition, other ongoing clinical trials focus on extending the indications of SGLT-2i and MR antagonism to a larger number of non-diabetic kidney disorders, such as patients with non-albuminuric CKD. Very recently, the EMPA-KIDNEY trial was designed to assess the effects of treatment with empagliflozin in a broad range of non-diabetic CKD patients. During a median of 2.0 years of follow-up, in a wide range of CKD patients, empagliflozin therapy led to a lower risk of progression of kidney disease or death from cardiovascular causes than placebo [8]. Therefore, current studies suggest a strong rationale for SGLT2 inhibition to be used into the standard of care for most CKD patients also with non-diabetic kidney disease.

While studying the implementation of the new guidelines, and trying to understand the new threshold for residual risk in DKD, a number of new treatments, derived from the basic research pipeline, are likely to be soon proposed. Recent progress in the understanding of mechanisms leading to CKD has shown that nephron loss is associated with inflammation, myofibroblast activation, tissue hypoxia, cell loss by apoptosis and senescence and extracellular matrix (ECM) deposition. Upregulated Nox expression and the decreased Nrf2 expression result in oxidative stress. The injured renal resident cells upregulate NLRP3 inflammasome, MAPK, PI3K/Akt and RAAS signaling and release proinflammatory chemokines to recruit immune cells such as macrophages from bone marrow which multiply the inflammatory effects. Targeting these pathways is expected to ameliorate outcomes and further extend basic and clinical investigation in CKD [9].

In addition to the recent breakthroughs in the treatment of disease progression, new science is being developed for the treatment of CKD complications. During its course, CKD is characterized by the progressive development of a series of complications, such as hypertension, left-ventricular hypertrophy (LVH) anemia, hyperkalemia, hypervolemia, hyperphosphatemia with mineral and bone disorders (CKD–MBD), metabolic acidosis, hyperuricemia and wasting; all of these complications have been shown to be associated with adverse outcomes, and can contribute either individually or in association to the morbidity and mortality observed in CKD. New treatments are available for many of these complications. One of the new concepts regards the importance of lifestyle modification strategies for primary and secondary CKD prevention. Maintaining a healthy body weight, physical activity, avoiding tobacco smoking, moderating alcohol consumption and healthy diets (low in sodium; rich in fruit, vegetables, and potassium; and with a higher plant-based to animal protein ratio) can prevent kidney disease [10]. Dietary and lifestyle modifications are also important for secondary care. A low-protein diet (LPD) has for many years been used to lower nitrogen (*N*)-derived catabolic products and delay uremic symptoms and in patients with CKD. Restriction in fruits and vegetables to prevent hyperkalemia has been one of the cornerstones of CKD nutritional approach. More recently, it has become clear that targeting patients’ nutrition is a necessary approach to prevent malnutrition, CKD complications and, possibly, disease progression. Recent cohort observational studies suggest that plant-dominant low-protein (PLADO) diets offer a better approach to nutritional treatment of CKD. The benefits of PLADO include increased fibre intake with reduced production of uraemic toxins by the gut microbiota, anti-atherogenic effects, better correction of metabolic acidosis and of CKD progression, and better control of hyperphosphataemia. The current concept is that restriction of plant foods as a strategy to prevent hyperkalaemia or undernutrition needs to be individualized to offer the benefit of PLADO without incurring hyperkalemia. However, proteins contained in plants are less anabolic than animal proteins and their constituent amino acids may be in part oxidized; therefore, amino acids contained in plant proteins may not completely be used for protein synthesis. Although PLADO has a preventive effect on the associated risk factors and influences CKD progression this anabolic potential may be low if used under protein-restricted regimens [11,12]. Therefore, research is needed to address the importance of diet-induced hyperkalaemia and nutritional status in CKD patients at advanced disease states.

Another new issue is on the treatment of metabolic acidosis. Small-scale trials in patients with CKD 3–5 have shown that hypobicarbonatemic metabolic acidosis promotes progression of CKD. Accordingly, the 2012 KDIGO guideline suggests base administration to patients with CKD when serum bicarbonate concentration is <22 mEq/L. However, recent observations in eubicarbonatemic patients with CKD suggest that also in these subjects, base administration ameliorates disease progression. If proven true, such knowledge would trigger a paradigm shift in the indication for alkali therapy in CKD [13].

Overall, these are only a few examples of the fast-moving issues in the treatment of CKD and of its complications. Several of the issues dealing with the prevention and treatment of CKD are addressed in this volume. We thank the MDPI staff for their leadership in producing this *Journal of Clinical Medicine* Special Issue. We hope the readers will enjoy and benefit from new insight.
